# Interpersonal satisfaction and classroom silence in Chinese universities: the mediating role of social anxiety

**DOI:** 10.3389/fpsyg.2026.1676791

**Published:** 2026-01-20

**Authors:** Yuqiang Liu, Mengzhu Liu, Siguang Zhang

**Affiliations:** 1 School of Economics and Management, North University of China, Taiyuan, China; 2 Institutes of Science and Development, Chinese Academy of Sciences, Beijing, China

**Keywords:** classroom silence, higher education, interpersonal satisfaction, psychological safety, social anxiety, student engagement

## Abstract

**Introduction:**

Within Chinese universities, classroom silence, while culturally acceptable, may indicate underlying psychological distress that impedes academic engagement. Drawing upon social-cognitive theory, this study investigates whether social anxiety mediates the relationship between interpersonal satisfaction and students’ in-class silence.

**Methods:**

A cross-sectional survey of 565 undergraduates from six Chinese universities examined interpersonal satisfaction, social anxiety, and three distinct behavioral indicators of silence (large-lecture, small-discussion, and laboratory contexts). Confirmatory factor analysis established convergent and discriminant validity; Cronbach’s *α* coefficients ranged from 0.86 to 0.91, demonstrating good internal consistency. A structural equation model (SEM) was employed to test the hypothesized mediation pathway while statistically controlling for gender, only-child status, hometown type, and personality traits.

**Results:**

The measurement model exhibited satisfactory fit indices (*χ*^2^/*df* = 2.34, CFI = 0.957, TLI = 0.948, RMSEA = 0.058, SRMR = 0.041). Within the structural model, interpersonal satisfaction demonstrated a significant negative prediction of social anxiety (*β* = −0.42, *p* < 0.001) and a comparatively weaker direct negative effect on silence (*β* = −0.18, *p* = 0.019). Social anxiety significantly and positively predicted silence (*β* = 0.51, *p* < 0.001) and mediated complete association, accounting for 54.5% of the total effect between satisfaction and silence (bootstrapped *β*_indirect = −0.21, 95% CI [−0.27, −0.15]). The model explained 18% of the variance in social anxiety and 39% of the variance in classroom silence.

**Discussion:**

Findings reveal a socio-emotional cascade: supportive relationships link to anxiety, which subsequently decreases the propensity for silence. The residual direct path indicates the presence of additional explanatory mechanisms. By delineating how relational climates associate with voice behavior, this study challenges essentialist attributions of Chinese students’ silence exclusively to Confucian deference norms. Due to cross-sectional design, causal inferences are tentative.

## 1 Introduction

Verbal participation in classroom interactions is widely acknowledged as a cornerstone of deep learning, critical thinking, and the cultivation of socio-academic competencies in higher education (Scanlon et al., 2020). Active engagement—manifested through questioning, responding, and contributing to discussions—facilitates knowledge construction and fosters students’ sense of belonging within academic communities. Yet, despite its pedagogical importance, a substantial proportion of university students, particularly in East Asian contexts, remain persistently silent during class. While silence can occasionally function as a culturally valued behavior signifying attentiveness and respect (Zhang and Zhang, 2018), a growing body of research indicates that prolonged or involuntary silence often reflects underlying psychological struggles, most notably social anxiety and dissatisfaction with interpersonal relationships, which are typically associated with lower academic engagement, weaker performance, and reduced overall well-being (Maher and King, 2022, 2023; Bi et al., 2022).

Social anxiety—defined as the persistent fear of negative evaluation by peers and authority figures, coupled with heightened physiological arousal and avoidance tendencies—has emerged as a primary emotional factor suppressing students’ willingness to speak in class (Lee et al., 2022). These fears generate cognitive distortions—such as catastrophizing minor errors and overestimating the likelihood of social rejection—which are associated with avoidance behaviors and more persistent classroom silence (Summerfeldt et al., 2006). Over time, this anxiety-driven silence not only deprives students of opportunities for skill development but also diminishes their academic self-efficacy, social integration, and future career readiness (Archbell and Robert J. Coplan, 2021; Scanlon et al., 2020).

In parallel, interpersonal satisfaction—the subjective evaluation of the quality, supportiveness, and inclusivity of students’ relationships with their peers and instructors—has gained attention as a distinct yet interrelated factor influencing classroom engagement. High interpersonal satisfaction is consistently associated with stronger senses of belonging and emotional security, which are in turn associated with lower evaluative apprehension and more active participation (Ma and Lin, 2022; Sikandar et al., 2023). Students embedded in supportive relational networks exhibit more frequent verbal contributions, higher emotional adjustment, and improved academic performance compared to those experiencing relational strain (De Souza Borba et al., 2020; Kahraman, 2022). Conversely, deficits in interpersonal satisfaction heighten feelings of social isolation and perceived vulnerability, both of which exacerbate anxiety-driven silence (Caballo et al., 2014). Consistent with Bandura’s (1986) social cognitive theory, interpersonal satisfaction is therefore positioned as a distal contextual antecedent rather than a covariate or moderator, shaping proximal emotional states—particularly social anxiety—that are closely related to students’ classroom participation.

Despite these advancements, significant conceptual and empirical gaps remain. Much of the extant literature has examined social anxiety and interpersonal satisfaction as separate predictors of engagement, neglecting their interactive and sequential effects on classroom silence (Maher and King, 2023; Noman and Xu, 2023). This oversight limits our understanding of the psychological mechanisms through which relational factors influence participation. Social-cognitive theory offers a robust conceptual framework to address these gaps, positing that environmental and relational contexts shape individuals’ emotional states, which in turn determine behavioral choices (Bandura, 1986). Applied to higher education, this suggests that interpersonal satisfaction functions as a contextual antecedent that influences social anxiety—a key emotional mediator—which directly governs students’ classroom engagement. Recent empirical evidence supports this model, demonstrating that relational dissatisfaction predicts heightened anxiety, which subsequently leads to diminished verbal participation (Archbell and Robert J. Coplan, 2021; Bi et al., 2022).

Furthermore, emerging research highlights the moderating and buffering roles of emotional intelligence (EI) and emotion regulation capacities within this process. Students with higher EI—characterized by enhanced emotional awareness, regulation, and interpersonal management—are better equipped to mitigate anxiety, sustain relational interactions, and engage actively despite psychosocial challenges (Kahraman, 2022; Zhao et al., 2024). Although EI is not a direct focus of the present study, acknowledging its role provides a broader context for interpreting how emotional competencies may influence the pathways linking interpersonal satisfaction, social anxiety, and classroom silence.

**Figure 1:**
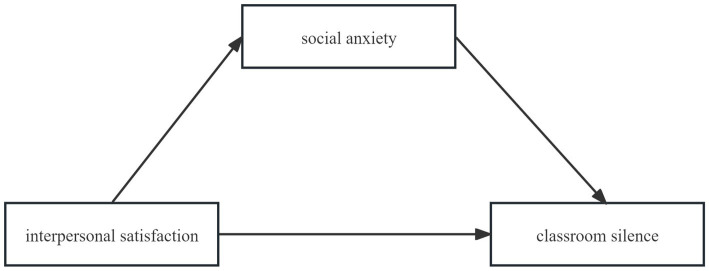
Hypothesized structural equation model.

Building on these theoretical and empirical considerations, the present study aims to advance understanding by systematically examining the mediating role of social anxiety in the association between interpersonal satisfaction and classroom silence among Chinese university students. We propose a conceptual model in which interpersonal satisfaction is hypothesized to be negatively associated with both social anxiety and silence, social anxiety is positively associated with silence, and social anxiety mediates the association between interpersonal satisfaction and silence. Figure 1 illustrates this hypothesized structural equation model. By situating this inquiry within the sociocultural realities of Chinese higher education—characterized by large class sizes, hierarchical pedagogies, and Confucian norms that both normalize and complicate silence—this research addresses critical gaps in prior scholarship. Ultimately, our findings aim to inform the design of evidence-based interventions and pedagogical strategies that reduce anxiety-related silence, foster inclusive learning environments, and promote holistic student development, while recognizing the constraints of a cross-sectional design.

Given these theoretical and empirical considerations, the present study seeks to address the following research questions: (1) to what extent is interpersonal satisfaction associated with university students’ classroom silence in the Chinese higher education context? (2) Does social anxiety function as a mediating mechanism in this association, and if so, how strongly does it account for the link between interpersonal satisfaction and classroom silence? Guided by the proposed theoretical framework and extant literature, we advance the following hypotheses:

*H1*: Interpersonal satisfaction is negatively associated with social anxiety, such that higher levels of interpersonal satisfaction correspond to lower levels of social anxiety.

*H2*: Social anxiety is positively associated with classroom silence, such that higher levels of social anxiety correspond to higher levels of classroom silence.

*H3*: Social anxiety mediates the relationship between interpersonal satisfaction and classroom silence, such that interpersonal satisfaction is indirectly associated with classroom silence through its relation to social anxiety.

## 2 Literature review

### 2.1 Social anxiety and classroom silence

Classroom silence has long been a focus of educational psychology, often interpreted as either a culturally normative behavior or a marker of disengagement. Classroom silence can be differentiated into voluntary/reflective silence, anxiety-driven silence, and functional silence. Voluntary or reflective silence refers to learners’ deliberate choice to remain quiet in order to think, observe, or consolidate information, and is often regarded as a productive learning strategy that supports cognitive processing and subsequent verbal participation (Hu, 2021). In contrast, anxiety-driven silence is a form of avoidance behavior rooted in fear, insecurity, or anticipated negative evaluation, which is typically associated with lower participation and fewer learning opportunities (Maher and King, 2020, 2023). Functional silence, which refers to the intentional use of silence by teachers or students to achieve specific educational goals, is acknowledged but not the primary focus of this research. Increasingly, however, research emphasizes that persistent, involuntary silence is frequently symptomatic of underlying psychological distress, particularly social anxiety (Chen et al., 2025). Social anxiety, characterized by excessive fear of negative evaluation, heightened physiological arousal, and avoidance of performance-related situations, exerts a profound influence on students’ willingness to participate verbally in class (Üstündağ et al., 2025). In university populations, social anxiety prevalence varies widely—ranging from 10 to 80%—but is notably higher in collectivist societies such as China, where cultural norms surrounding “face,” conformity, and respect intensify evaluative concerns (Han et al., 2025).

Empirical studies consistently document the disruptive effects of social anxiety on academic engagement. Students with elevated anxiety frequently avoid speaking to prevent perceived embarrassment or rejection, even when cognitively prepared to contribute (Almadina Rakhmaniar, 2023; Archbell and Robert J. Coplan, 2021). This avoidance is compounded by cognitive distortions such as catastrophizing potential mistakes, overestimating the likelihood of negative peer judgment, and internalizing neutral cues as criticism (Li X. et al., 2024; Summerfeldt et al., 2006). These maladaptive thought patterns reinforce avoidance, perpetuating a self-sustaining cycle of silence and disengagement (Scanlon et al., 2020). Over time, such behaviors erode students’ confidence, academic performance, and social connectedness, with implications that extend beyond university into professional and personal domains (Lu et al., 2025).

The sociocultural context of East Asian classrooms magnifies these dynamics. Chinese university students often navigate large class sizes, hierarchical instructor-student relationships, and competitive academic environments, all of which amplify perceived risks associated with verbal participation (Xu et al., 2022). In such contexts, silence is sometimes valorized as a sign of discipline or attentiveness, yet evidence shows that psychological drivers such as anxiety—not cultural norms alone—explain much of the observed reticence (Chan and Smith, 2024; Langen and Stamov Roßnagel, 2023). These findings highlight the need for context-sensitive research disentangling voluntary, culturally normative silence from involuntary, anxiety-driven disengagement.

### 2.2 Interpersonal satisfaction and classroom engagement

Beyond emotional vulnerabilities, the quality of students’ interpersonal relationships—captured through the construct of interpersonal satisfaction—plays a pivotal role in shaping classroom behaviors. Interpersonal satisfaction encompasses students’ perceptions of the quality, supportiveness, and inclusivity of their relationships with peers and instructors. High interpersonal satisfaction fosters emotional safety, strengthens belonging, and mitigates evaluative fears, all of which encourage active engagement (Chen et al., 2023; Kang et al., 2025). Research consistently links supportive relational environments with increased verbal contributions, improved emotional adjustment, and enhanced academic performance (De Souza Borba et al., 2020; Kahraman, 2022).

Conversely, relational dissatisfaction correlates with heightened feelings of isolation, exclusion, and vulnerability, which exacerbate students’ reluctance to speak and heighten their susceptibility to social anxiety (Caballo et al., 2014). Students perceiving their interpersonal environment as unsupportive often interpret classroom interactions as threatening, triggering avoidance behaviors that further hinder participation (Bauth et al., 2019; Liu et al., 2024). Interpersonal competencies—such as empathy, assertiveness, and conflict resolution—further influence these dynamics. Students with well-developed social skills are more adept at navigating peer and instructor relationships, report lower anxiety, and engage more readily in classroom discourse (Bolsoni-Silva and Loureiro, 2014; Summerfeldt et al., 2006).

Interpersonal satisfaction is prioritized over other relational constructs such as peer belonging and teacher immediacy because it directly captures the emotional quality of interactions in educational settings. While peer belonging focuses on the sense of group inclusion and acceptance (McBeath et al., 2018), and teacher immediacy examines the teacher’s behaviors to reduce psychological distance, interpersonal satisfaction provides a more comprehensive measure of the emotional dynamics in both teacher-student and student–student interactions (Dos Santos et al., 2019).

The literature underscores that fostering supportive, inclusive classroom climates is a structural strategy to counteract both relational dissatisfaction and its downstream effects on anxiety and silence. Such efforts not only enhance individual student outcomes but also contribute to more dynamic, interactive learning environments.

### 2.3 The mediating role of social anxiety

Although the literature has robustly established the independent effects of social anxiety and interpersonal satisfaction on student engagement, research integrating these constructs remains comparatively sparse. Emerging studies propose that social anxiety may serve as a key mediating mechanism through which deficits in interpersonal satisfaction translate into classroom silence (Xethakis et al., 2024; Demir et al., 2023). Students embedded in unsupportive relational networks often experience heightened evaluative threat, which triggers anxiety that directly suppresses verbal participation (Mi et al., 2025).

This mediational framework aligns with Bandura's (1986) social-cognitive theory, which posits that environmental stimuli influence emotional states, which subsequently govern behavior. Within this framework, interpersonal satisfaction functions as a contextual antecedent shaping students’ emotional responses—specifically social anxiety—which in turn dictate engagement patterns. Recent findings lend empirical support to this sequential model, suggesting that interventions targeting relational contexts may indirectly reduce silence by alleviating anxiety (Ma and Xia, 2025).

Emotional intelligence (EI) research further contextualizes this process. Students with stronger EI—marked by heightened emotional awareness, regulation, and management skills—are better positioned to navigate relational stressors, mitigate anxiety, and sustain participation (Choi, 2025). While EI is not the central focus of the present study, recognizing its buffering role underscores the multifaceted emotional processes shaping the satisfaction–anxiety–silence pathway.

### 2.4 Intervention and pedagogical implications

Conceptualizing social anxiety as a mediator yields practical implications for pedagogy and student support. A growing body of evidence indicates that interventions targeting both emotional and relational dimensions can effectively reduce silence and promote engagement. Socio-emotional learning (SEL) programs, interactive teaching strategies, and cognitive-behavioral techniques have shown promise in alleviating anxiety and in fostering supportive classroom environments (Hassani, 2024; Guo et al., 2025).

Instructors play a pivotal role in these efforts. Teaching practices that prioritize constructive feedback, model inclusive participation norms, and actively cultivate relational trust have been shown to reduce student anxiety and increase verbal contributions (König et al., 2022; Ai et al., 2025). Institutional initiatives to train educators in emotional sensitivity and relational skill-building thus represent essential components of a comprehensive strategy to address anxiety-driven silence.

### 2.5 Gaps and future research directions

Despite these advances, several gaps remain. First, few studies employ longitudinal or cross-institutional designs capable of capturing the dynamic interplay between interpersonal satisfaction, social anxiety, and classroom silence over time (Li and Li, 2023; Xu and Zhou, 2024). Second, methodological inconsistencies—particularly in operationalizing constructs like silence and interpersonal satisfaction—limit theoretical coherence and comparability across studies (Myoung and Liou, 2025). Third, research remains heavily concentrated in single-institution or Western contexts, limiting the generalizability of findings to diverse cultural and educational environments (Chen et al., 2025).

Future research should adopt standardized assessment tools, longitudinal frameworks, and culturally diverse samples to clarify these mediational processes. Such efforts would not only advance theoretical understanding but also inform the development of culturally sensitive interventions capable of addressing the intertwined emotional and relational drivers of classroom silence in Chinese and broader East Asian higher education contexts.

## 3 Methodology

### 3.1 Research design

This study employed a quantitative, cross-sectional survey design to examine the mediating role of social anxiety in the association between interpersonal satisfaction and classroom silence among undergraduate students in Chinese universities. A structured questionnaire allowed for standardized assessment of latent constructs and their interrelations across a relatively diverse student sample. This design is consistent with prior work on classroom silence and socio-emotional factors, which has demonstrated the utility of cross-sectional approaches for capturing psychological correlates of student engagement in higher education contexts (Maher and King, 2022; Noman and Xu, 2023).

To test the hypothesized mediational framework, structural equation modeling (SEM) was chosen as the primary analytical technique. SEM is well suited to this study because it simultaneously estimates measurement and structural models, allows for the control of measurement error, and enables formal testing of indirect effects. This approach has been widely adopted in recent research examining the psychological and relational predictors of student engagement and silence (Yang et al., 2024; Xu and Zhou, 2024).

### 3.2 Participants

Participants were drawn from six universities located in Taiyuan, Shanxi Province, China. All six institutions are public, teaching-oriented comprehensive universities rather than elite research-intensive (“Double First-Class”) campuses, enrolling predominantly undergraduate and applied master’s students and serving regional talent development missions typical of many second-tier Chinese universities.

To enhance representativeness within this single-city context, a stratified multistage convenience sampling strategy was adopted. First, faculties/schools within each university were stratified by disciplinary cluster (humanities and social sciences, natural sciences, engineering/technology, and management/business). Second, classes within each stratum were approached through course instructors and student counselors. Finally, individual students were invited to voluntarily complete the survey. Data were collected between June and July 2025.

Following recommended guidelines for SEM (i.e., ≥5–10 cases per observed indicator; Bentler and Chih-Ping Chou, 1987), we targeted a minimum of 400 respondents. The final usable sample comprised 565 undergraduate students, aged 18–24 years. Respondents reported demographic information including academic major, year of study (1 = Freshman, 2 = Sophomore, 3 = Junior, 4 = Senior), gender (0 = Male, 1 = Female), only-child status (0 = No, 1 = Yes), and hometown type (1 = Rural, 2 = Urban, 3 = Town).

All participation was voluntary and anonymous. Before starting the survey, participants read an online information sheet describing the study purpose, confidentiality safeguards, and their right to withdraw at any time without penalty, and then provided electronic informed consent. No identifying personal information (e.g., name, student ID, IP address) was collected. According to local institutional guidelines and national regulations, anonymous, non-interventional questionnaire studies are exempt from formal ethical review; therefore, no institutional ethics approval was required for this research.

### 3.3 Measures

The questionnaire comprised four core sections: (a) demographic and personality control variables, (b) classroom silence, (c) social anxiety, and (d) interpersonal satisfaction. Unless otherwise specified, non-demographic items were rated on five-point Likert-type scales ranging from 1 (strongly disagree) to 5 (strongly agree). All multi-item measures were adapted from validated scales in the existing literature. Following best practices for cross-cultural adaptation (Brislin, 1970), all items underwent translation and back-translation procedures. Two bilingual experts in educational psychology independently translated the original English items into Chinese. An independent third translator, blind to the originals, then back-translated the Chinese versions into English. Discrepancies were discussed and resolved by the research team to ensure linguistic and conceptual equivalence.

A pilot study with 50 undergraduate students was conducted to refine item wording and assess preliminary reliability. Pilot participants provided qualitative feedback on clarity and relevance, leading to minor wording adjustments for several items. Preliminary analyses indicated acceptable internal consistency (Cronbach’s *α* ≥ 0.70) for the key scales, supporting their suitability for use in the main study. An overview of all observed variables, item descriptions, and coding schemes is provided in Table 1.

**Table 1:** Data coding and variable definitions.

Variable name	Item description	Data type	Response options	Numerical coding
major	Academic major	Text	Major name	Original text
year_of_study	Academic year	Ordinal	Freshman/Sophomore/Junior/Senior	1 = Freshman, 2 = Sophomore, 3 = Junior, 4 = Senior
gender	Gender	Binary	Male/Female	0 = Male, 1 = Female
is_only_child	Only child status	Binary	Yes/No	0 = No, 1 = Yes
hometown	Type of hometown	Categorical	Rural/Urban/Town	1 = Rural, 2 = Urban, 3 = Town
large_class_speak	Frequency of speaking in large lectures	Ordinal	Never/1–2 times/3–5 times/5 + times	1–4
small_class_speak	Frequency of speaking in small discussions	Ordinal	Same as above	1–4
lab_class_speak	Frequency of speaking in lab classes	Ordinal	Same as above	1–4
silence_teacher	Silent when called by teacher	Binary	Selected/Not selected	0 = Not selected, 1 = Selected
silence_discussion	Silent during open discussion	Binary	Selected/Not selected	0/1
silence_presentation	Silent during group presentations	Binary	Selected/Not selected	0/1
silence_difficult	Silent when faced with difficult questions	Binary	Selected/Not selected	0/1
personality_sociable	Outgoing and sociable	Likert scale (5)	Strongly disagree to Strongly agree	1–5
personality_nervous	Easily nervous	Likert scale (5)	Same as above	1–5
personality_empathetic	Empathetic	Likert scale (5)	Same as above	1–5
personality_organized	Organized	Likert scale (5)	Same as above	1–5
anxiety_performance	Anxious about performance in social situations	Likert scale (5)	Same as above	1–5
anxiety_eye_contact	Nervous when others look at me	Likert scale (5)	Same as above	1–5
anxiety_stranger	Uncomfortable communicating with strangers	Likert scale (5)	Same as above	1–5
anxiety_public_speak	Anxious about public speaking	Likert scale (5)	Same as above	1–5
anxiety_social_event	Uneasy before attending social events	Likert scale (5)	Same as above	1–5
relationship_dorm	Satisfaction with dorm relationships	Likert scale (5)	Same as above	1–5
relationship_classroom	Good relationship with classmates	Likert scale (5)	Same as above	1–5
relationship_teacher	Comfortable relationship with teachers	Likert scale (5)	Same as above	1–5
relationship_friend	Have friends to confide in	Likert scale (5)	Same as above	1–5
relationship_positive	Relationships positively influence learning	Likert scale (5)	Same as above	1–5

#### 3.3.1 Demographic and control variables

The demographic variables collected and coded for analysis included academic major, year of study, gender, only-child status, and hometown type, as described in the Participants section. To account for personality-related confounding factors, four personality traits were measured using single Likert items adapted from the Big Five Inventory (John et al., 2008): sociability (“I am outgoing and sociable”) from Extraversion, nervousness (“I tend to become nervous easily”) from Neuroticism, empathy (“I am empathetic towards others”) from Agreeableness, and organization (“I am organized and systematic”) from Conscientiousness. These variables were included as coarse controls to help isolate the unique associations of interpersonal satisfaction and social anxiety with classroom silence. Because each trait was assessed with a single item rather than a multi-item scale, they should be interpreted as approximate indicators of broad dispositions rather than fully reliable latent constructs; this limitation is revisited in the discussion. Descriptive statistics for the demographic and personality covariates are reported in Table 2.

**Table 2:** Descriptive statistics of demographic and personality covariates (*N* = 565).

Variable	Min	Max	M	SD	Median
Grade	1.00	4.00	2.10	1.00	2.00
Gender (0 = Male, 1 = Female)	0.00	1.00	0.51	0.50	1.00
Only_child	0.00	1.00	0.46	0.50	0.00
Personality_sociable	1.00	5.00	3.87	1.35	4.00
Personality_nervous	1.00	5.00	3.52	1.29	4.00
Personality_empathetic	1.00	5.00	3.95	1.23	4.00
Personality_organized	1.00	5.00	3.58	1.24	4.00

Gender and only-child status are binary-coded variables (0/1), therefore means represent proportions. Personality traits were measured with single-item indicators adapted from the Big Five Inventory.

#### 3.3.2 Classroom silence

Classroom silence was operationalized using both frequency-based measures and context-specific indicators. Students reported their typical frequency of verbal participation in three instructional formats: large lectures, small-group discussions, and laboratory sessions, using a four-point ordinal scale (1 = Never, 2 = 1–2 times, 3 = 3–5 times, 4 = More than 5 times). Additionally, four binary items captured silence behaviors in specific contexts: (1) remaining silent when directly called upon by the instructor, (2) refraining from speaking during open class discussions, (3) avoiding verbal contributions during group presentations, and (4) staying silent when confronted with difficult or unexpected questions. Together, these seven indicators were intended to reflect students’ overall propensity to remain silent across instructional formats and evaluative situations. Conceptually, the frequency-based and binary indicators were combined under a single latent construct because they were designed to represent a common underlying disposition toward classroom silence, rather than distinct subtypes of behavior. Psychometrically, confirmatory factor analysis (CFA) supported this unidimensional specification: all seven items loaded significantly and substantially on a single factor, and the one-factor model showed good fit (e.g., CFI ≈ 0.95, RMSEA ≈ 0.06) and outperformed a two-factor model that separated frequency and binary items (ΔCFI < 0.01). On this basis, classroom silence was modeled as a unidimensional latent variable in the SEM analyses. At the same time, we acknowledge that this aggregate approach cannot fully differentiate reflective or strategic quiet from anxiety-driven silence; this limitation is discussed further below.

#### 3.3.3 Social anxiety

Social anxiety was assessed using five items adapted from validated scales commonly used in higher education research. Items tapping anxiety about performing poorly in social or evaluative situations, public speaking anxiety, and anticipatory anxiety before social events were adapted from the social anxiety items used by Lee et al. (2022), which emphasizes fear of negative evaluation in academic performance contexts. Items assessing discomfort during sustained eye contact (“I feel nervous when others look at me”) and unease when communicating with strangers were adapted from (Maher and King, 2023), which focuses on interactional anxiety in classroom communication. Together, these five items were intended to capture core manifestations of social anxiety that are particularly salient in university students’ academic lives. Participants rated the extent to which each statement described them on a five-point scale (1 = strongly disagree, 5 = strongly agree). Prior studies using similar item sets have reported high internal consistency (Cronbach’s *α* ≥ 0.85) and strong associations with classroom avoidance and withdrawal (Bi et al., 2022). In the present sample, the five-item social anxiety scale also demonstrated satisfactory reliability and unidimensionality (see Table 3).

**Table 3:** Descriptive statistics, reliabilities, and correlations (*N* = 565).

Variable	M	SD	α/CR	1	2	3
Interpersonal satisfaction (IS)	3.71	0.62	0.88/0.89	–		
Social anxiety (SA)	3.42	0.68	0.90/0.91	−0.45***	–	
Classroom silence (CS)	2.31	0.79	0.86/0.87	−0.29***	0.52***	–

α = Cronbach’s alpha; CR = composite reliability. All correlations are Pearson’s r. ***p < 0.001.

#### 3.3.4 Interpersonal satisfaction

Interpersonal satisfaction was measured using five items assessing perceived relational quality across key social contexts in university life. Items were adapted primarily from relationship quality scales used in student well-being and engagement research, including the Quality of Relationships Inventory (QRI) framework as applied by Collie et al. (2016) and relational engagement items from Sikandar et al. (2023). The selected contexts—relationships with dormitory peers, classroom peers, teachers, and close friends—reflect domains emphasized in the literature as central to students’ academic experience and psychological adjustment (Chen et al., 2023; Kang et al., 2025).

Items assessed satisfaction with dormitory relationships (“I am satisfied with dormitory relationships”), classroom peer interactions (“I get along well with classmates”), teacher–student relationships (“I feel comfortable with my teachers”), access to supportive friendships (“I have friends to confide in”), and the perceived positive influence of relationships on academic outcomes (“My interpersonal relationships positively influence my studies”). Responses ranged from 1 (strongly disagree) to 5 (strongly agree). Previous studies report high internal consistency for similar item sets (Cronbach’s α = 0.85–0.91) and robust predictive validity for student engagement and well-being (Collie et al., 2016; Sikandar et al., 2023). In the present study, the interpersonal satisfaction scale also exhibited good internal consistency and convergent validity (see Table 3).

### 3.4 Data collection procedure

Data were collected over a four-week period via an online survey hosted on the Wenjuanxing (WJX) platform, a widely used survey tool in China. Recruitment utilized academic social media groups, online student forums, and classroom announcements coordinated with participating instructors. To ensure ethical integrity, participation was strictly voluntary and anonymous. The first page of the survey presented an information and consent form explaining the study’s purpose, the anonymous and confidential handling of data, and participants’ right to withdraw at any time without adverse consequences. Students indicated their consent by clicking an “I agree to participate” button before accessing the questionnaire. No identifying information (e.g., name, student ID, IP address) was recorded. Data quality was ensured through multiple procedures: (1) screening for incomplete or logically inconsistent responses, (2) removal of cases with abnormally short completion times, and (3) inclusion of an attention-check item (“Select ‘agree’ for this statement to confirm your attention”). After cleaning, the final sample met all inclusion criteria and exhibited no significant demographic biases relative to the initial recruitment pool.

### 3.5 Data coding and variable definitions

All variables were coded systematically for analysis, with coding schemes detailed in Table 1. Ordinal Likert-type items with four or five response categories (e.g., indicators of social anxiety, interpersonal satisfaction, and classroom silence frequency) were treated as continuous in the SEM analyses. Following Rhemtulla et al. (2012), when items have at least four categories and approximately symmetric distributions, robust continuous estimators perform comparably to categorical estimators in terms of bias and efficiency. Preliminary inspection of the present data indicated no severe skewness or floor/ceiling effects for the focal constructs, supporting this analytic choice.

Personality control items, social anxiety indicators, and interpersonal satisfaction items were coded so that higher scores indicated higher levels of the underlying constructs. For descriptive purposes, composite scores were computed as the mean of the relevant items for each scale.

### 3.6 Data analysis approach

Data analysis proceeded in four stages. First, descriptive statistics were computed to summarize participant demographics and core study variables (see Tables 2, 3). Second, confirmatory factor analysis (CFA) was conducted to validate the measurement model, ensuring that the latent constructs (classroom silence, social anxiety, interpersonal satisfaction) exhibited satisfactory reliability (Cronbach’s *α* ≥ 0.70) and convergent validity (average variance extracted ≥ 0.50). Third, the hypothesized structural model was estimated using SEM, testing direct and indirect effects, with bootstrapped confidence intervals (5,000 resamples) employed to assess the statistical significance of mediational pathways. Finally, competing models (e.g., alternative ordering of IS, SA, and CS) were explored in supplementary analyses to assess the robustness of the hypothesized structure.

Model fit was evaluated using multiple indices: the Comparative Fit Index (CFI) and Tucker–Lewis Index (TLI) with thresholds ≥0.90 for acceptable fit, the Root Mean Square Error of Approximation (RMSEA) with values ≤0.08, and the Standardized Root Mean Square Residual (SRMR) with values ≤0.08 (Hu and Bentler, 1999). All analyses were conducted using SPSS 28.0 for descriptive and preliminary analyses and AMOS 28.0 for CFA and SEM estimation.

## 4 Results

### 4.1 Data screening and preliminary diagnostics

Of the 612 questionnaires returned, 47 were excluded because of excessive missing data (>20%), straight-lining, or failure on the attention-check item. The final analytic sample comprised 565 undergraduates (50.97% female; Mage = 20.1, *SD* = 1.32) from six universities. Little’s MCAR test was non-significant (*χ*^2^ = 124.37, *df* = 118, *p* = 0.32), suggesting data were missing completely at random; remaining sporadic missing values (<1%) were imputed using expectation–maximization. Variance inflation factors (VIFs < 2.30) indicated no problematic multicollinearity. A Harman single-factor test (first factor = 28.4%) and an unmeasured latent-method factor model (ΔCFI = 0.004) suggested common-method bias was not a serious concern, although some degree of shared-method variance cannot be entirely ruled out.

### 4.2 Descriptive statistics and correlations

Tables 2, 3 report means, standard deviations, reliabilities, and latent-variable correlations. On average, students’ self-reported scores on the focal variables were as follows: social anxiety (*M* = 3.42, *SD* = 0.68), interpersonal satisfaction (*M* = 3.71, *SD* = 0.62), and classroom silence (*M* = 2.31, *SD* = 0.79). We therefore focus on describing the observed score distributions rather than assigning qualitative labels (e.g., “low,” “moderate”) to these levels, given the absence of established clinical cutoffs for these adapted scales. Internal consistency was strong for all latent constructs (*α* = 0 0.86–0.91). Interpersonal satisfaction correlated negatively with social anxiety (*r* = −0.45, *p* < 0.001) and classroom silence (*r* = −0.29, *p* < 0.001), while social anxiety correlated positively with classroom silence (*r* = 0.52, *p* < 0.001).

### 4.3 Measurement model: reliability and validity

A confirmatory factor analysis (CFA) evaluated the three-factor measurement model—interpersonal satisfaction (IS), social anxiety (SA), and classroom silence (CS). The model demonstrated good fit, *χ^2^* (167) = 391.24, χ^2^/*df* = 2.34, CFI = 0.957, TLI = 0.948, RMSEA = 0.058 (90% CI [0.049, 0.066]), SRMR = 0.041. Standardized factor loadings ranged from 0.63 to 0.88 (all *p* < 0.001), supporting indicator reliability. Convergent validity was confirmed by Average Variance Extracted values of 0.56 (IS), 0.59 (SA), and 0.54 (CS), each exceeding the 0.50 benchmark. Discriminant validity held: the square roots of AVEs surpassed the corresponding inter-construct correlations, and Heterotrait–Monotrait ratios ranged from 0.39 to 0.71, well below the 0.85 cutoff. Detailed loadings are summarized in Table 4.

**Table 4:** Confirmatory factor analysis results.

Construct/Item	Loading
Interpersonal Satisfaction (IS)	
IS1 Dormitory relationships	0.78
IS2 Classmate relations	0.81
IS3 Teacher–student relations	0.74
IS4 Supportive friends	0.83
IS5 Positive academic impact	0.68
Social Anxiety (SA)	
SA1 Performance worry	0.85
SA2 Eye-contact nervousness	0.74
SA3 Stranger interaction discomfort	0.79
SA4 Public speaking anxiety	0.88
SA5 Pre-event uneasiness	0.71
Classroom Silence (CS)	
CS1 Large-lecture silence (freq.)	0.63
CS2 Small-discussion silence (freq.)	0.72
CS3 Lab-class silence (freq.)	0.70
CS4 Silent when called by teacher	0.77
CS5 Silent during open discussion	0.81
CS6 Silent in group presentation	0.76
CS7 Silent when questions are difficult	0.69

All loadings *p* < 0.001. AVE/CR: IS (0.56/0.89); SA (0.59/0.91); CS (0.54/0.87).

### 4.4 Structural model and hypothesis testing

**Figure 2:**
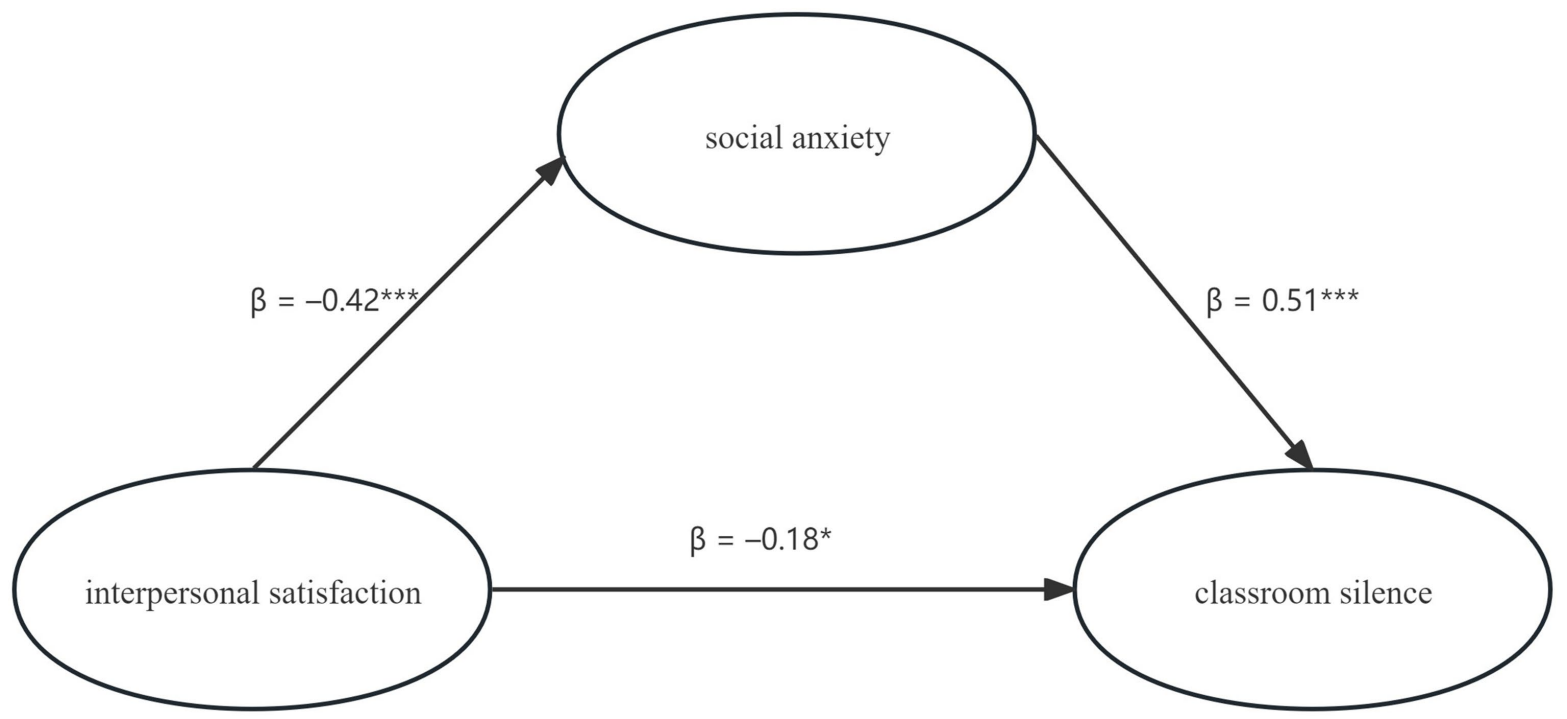
Standardized solution of the SEM.

The hypothesized mediation model (Figure 1) demonstrated good global fit—χ^2^ (169) = 402.11, χ^2^/*df* = 2.38, CFI = 0.956, TLI = 0.947, RMSEA = 0.059, SRMR = 0.043—after accounting for the control variables (sociability, nervousness, gender, only-child status, hometown type), none of which altered the significance or strength of the focal paths. Figure 2 presents the standardized solution, while Table 5 summarizes all structural coefficients and bootstrapped mediation statistics.

**Table 5:** Structural path estimates and mediation test (standardized coefficients).

Path	β	SE	95% CI	*p*
IS → SA	−0.42	0.05	[−0.52, −0.33]	< 0.001
SA → CS	0.51	0.06	[0.40, 0.62]	< 0.001
IS → CS (direct)	−0.18	0.07	[−0.32, −0.03]	0.019
Indirect (IS → SA → CS)	−0.21	0.04	[−0.27, −0.15]	< 0.001

Bootstrapped 95% CIs (5,000 resamples). IS = Interpersonal Satisfaction; SA = Social Anxiety; CS = Classroom Silence.

#### 4.4.1 Direct associations

As shown in Table 5, interpersonal satisfaction was negatively associated with social anxiety (*β* = −0.42, SE = 0.05, *p* < 0.001) and showed a smaller, yet statistically significant, negative association with classroom silence (*β* = −0.18, SE = 0.07, *p* = 0.019). Social anxiety, in turn, was positively associated with classroom silence (*β* = 0.51, SE = 0.06, *p* < 0.001).

#### 4.4.2 Indirect (mediated) association

Bias-corrected bootstrapping with 5,000 resamples confirmed a significant indirect pathway from interpersonal satisfaction to classroom silence through social anxiety (*β*_indirect = −0.21, 95% CI [−0.27, −0.15]; see Table 5). This indirect pathway accounted for 54.5% of the total effect (|*β*_indirect| / |*β*_total|). The overall effect of interpersonal satisfaction on silence remained significant (*β*_total = −0.39, *p* < 0.001), a pattern consistent with the proposed mediational hypothesis (H3), while still being interpreted in correlational rather than strictly causal terms.

#### 4.4.3 Explained variance

The model accounted for 18% of the variance in social anxiety (R^2^_SA = 0.18) and 39% in classroom silence (R^2^_CS = 0.39), indicating that the proposed variables capture meaningful, though not exhaustive, proportions of outcome variability.

### 4.5 Robustness and supplementary analyses

#### 4.5.1 Alternative models

Two rival models were tested: (a) a partial mediation model with an added path SA → IS (reverse causality), and (b) a non-mediation model omitting the indirect pathway. Both rivals fit significantly worse (ΔCFI >0.010; Δχ^2^ *p* < 0.01), suggesting that, within the limitations of cross-sectional data, the proposed ordering (IS → SA → CS) provides a more parsimonious representation of the observed covariance structure.

#### 4.5.2 Multi-group invariance

To explore potential gender differences, a multi-group SEM compared constrained and unconstrained models. Configural, metric, and scalar invariance were all supported (ΔCFI ≤ 0.005), indicating that the measurement and structural relations were comparable across male and female students. Similar invariance held across academic year groups (freshman vs. senior).

#### 4.5.3 Common method variance check

A common latent factor accounted for an additional 6.8% of variance; model fit improvement was negligible (ΔCFI = 0.003), suggesting that common method variance is unlikely to bias the substantive findings, although it may contribute modestly to the size of some associations.

## 5 Discussion

### 5.1 Synthesizing and deepening the core findings

Taken together, the structural model yielded a pattern of associations that is consistent with our hypothesized framework. The significant negative path from interpersonal satisfaction to social anxiety, and the positive path from social anxiety to classroom silence, were in line with H1 and H2, respectively, while the combination of direct and indirect paths between interpersonal satisfaction and silence aligned with H3. The SEM results therefore point to a coherent socio-emotional pathway from interpersonal satisfaction (IS) to classroom silence (CS) through social anxiety (SA), while also revealing a modest direct effect of IS on CS. The negative, medium-to-large standardized path from IS to SA (*β* = −0.42) indicates that students’ perceptions of supportive peer and instructor relationships are associated with dampened fears. This accords with the broader engagement literature showing that relational security anchors students’ willingness to take academic risks (Sun et al., 2024). The positive, large effect of SA on CS (*β* = 0.51) underscores anxiety’s proximal force in shaping communicative behavior, corroborating work in language and general education contexts where fear of negative evaluation is the strongest single predictor of verbal withdrawal (Archbell and Robert J. Coplan, 2021).

The small yet significant direct path from IS to CS (*β* = −0.18) suggests that relationships are related to classroom silence beyond their association with anxiety reduction. Several non-affective mechanisms may account for this residual effect (Jia and Cheng, 2024). First, supportive networks likely increase instrumental motivation to contribute—students may speak to reciprocate help or fulfill implicit group norms (Noman and Xu, 2023). Second, IS may foster perceived task value—students in positive relational climates often experience discussions as collaborative meaning-making rather than performance trials (Kelly et al., 2024). Third, positive relational climates may subtly reshape classroom norms, normalizing imperfection and reframing mistakes as shared learning opportunities, thereby reducing silence even when residual anxiety persists (Sikandar et al., 2023).

Variance explained (R^2^_SA = 0 0.18; R^2^_CS = 0.39) reveals that while our model captures substantial portions of the outcome variability, a sizable share remains unaccounted for—consistent with the multifactorial nature of participation. Factors such as dispositional introversion, prior negative experiences, language proficiency, or task-specific self-efficacy likely contribute (Chen et al., 2025; Özdemir and Seçkin, 2025; Sezin and Sal, 2025). Moreover, given the cross-sectional nature of the data, the directionality of these associations should be interpreted cautiously; it remains plausible that changes in speaking behavior also shape subsequent interpersonal satisfaction and anxiety levels.

### 5.2 Theoretical contributions

#### 5.2.1 A layered socio-emotional model of silence

The first theoretical advance is the articulation of a layered model in which IS functions as a distal relational antecedent, SA as a proximal affective mechanism, and CS as the behavioral outcome. Prior research has often pitted “culture” against “personality” or “anxiety” against “belonging” to explain silence (Zhang and Zhang, 2018). Our findings bridge these strands by specifying a sequential flow—relationships influence emotions, which shape behavior—thereby offering a clarifying synthesis rather than a competitive dichotomy. This layered view also helps explain discrepant cases observed qualitatively (Maher and King, 2022; Xethakis et al., 2024): some students report strong friendships yet still refrain from speaking (high IS, high SA); others report anxiety but remain vocal in classes perceived as safe (moderate SA, but very high IS).

#### 5.2.2 Operationalizing social-cognitive theory in higher education

Second, by empirically grounding the environment–person–behavior triad, we refine social-cognitive accounts of academic participation (Bandura, 1986). Our model operationalizes “environment” as perceived interpersonal quality, “person” as anxiety states, and “behavior” as speaking/remaining silent. The partial mediation is compatible with the possibility of bidirectional influences—students who voice more may accumulate relational capital and see anxiety fall, while chronic silence may degrade relationships and elevate anxiety. Although our cross-sectional design cannot test these dynamic feedback processes directly, the present pattern of associations underscores the value of longitudinal and cross-lagged investigations (Bittel et al., 2023; Scanlon et al., 2020; Jaffee et al., 2012).

#### 5.2.3 Re-centering psychosocial mechanisms within cultural frames

Third, we nuance cultural explanations of Chinese classroom silence. Rather than dismissing culture, we reposition it as a macro-context that normalizes silence, making it a socially permissible coping strategy. Yet, the immediate engine of silence for many students appears psychological—anchored in anxiety—not simply normative deference (Bi et al., 2022; Zou, 2024; Xethakis et al., 2024). This perspective encourages scholars to interrogate the interaction between cultural affordances and individual affective states, avoiding essentialist readings that reduce East Asian learners to “silent by tradition.”

#### 5.2.4 Cultural background as a contextual moderator

Building on self-construal theory (Markus and Kitayama, 1991), the IS–SA association may be particularly pronounced in collectivist, interdependent cultural contexts, where psychological well-being is closely tied to perceived relational harmony. Studies of East Asian and Chinese students have shown that interpersonal strain tends to be more strongly linked to anxiety symptoms than in more individualistic settings (Okazaki, 1997; Liu and Jackson, 2008). In this sense, our findings are consistent with the idea that, in cultures that place a high premium on relationships and “face,” higher interpersonal satisfaction is more likely to be associated with lower social anxiety, which in turn is associated with reduced anxiety-driven silence. Future cross-cultural work is needed to test whether the strength of the IS–SA–CS pathways varies across cultural and institutional contexts.

### 5.3 Mechanisms and boundary conditions

At the cognitive level, relational support offers corrective information that disrupts maladaptive appraisals, diminishing threat perception (Li X. et al., 2024). Affective pathways involve co-regulation: empathy, validation, and emotional contagion within supportive networks help students down-regulate arousal before it escalates into avoidance (Wang et al., 2025; Kahraman, 2022). Motivationally, belonging anchors identity: speaking becomes a contribution to the group rather than a solo performance, reframing the cost–benefit calculus (De Souza Borba et al., 2020). Socially, IS may change perceived norms—if trusted peers model question-asking and teachers respond supportively, silence loses its protective value (Krull et al., 2022).

Conversely, SA propels silence through attentional narrowing (hypervigilance to negative cues), working-memory taxation (rumination crowds out processing resources), and somatic feedback loops (interpreting physiological arousal as evidence of incompetence) (Larrazabal et al., 2025; Wang et al., 2024; Phylactou et al., 2024; Summerfeldt et al., 2006). These mechanisms may be intensified in evaluative contexts (presentations, cold-calling) and large classes where anonymity paradoxically raises fear of public error (Yep et al., 2023; Li B. et al., 2024).

Boundary conditions emerge when we consider individual differences and contextual moderators. High EI students might still speak under low IS because they can self-regulate anxiety (Pentón Herrera, 2024; Babanoğlu, 2025); similarly, even high IS may not eliminate silence if instructors use punitive grading or ridicule (Hu and Wang, 2023). These contingencies highlight that the observed pathways should be interpreted as average tendencies rather than deterministic rules, and they point toward future moderated-mediation and multilevel models.

### 5.4 Practical implications

The findings imply that effective interventions may need to work on both sides of the pathway: the relational ecology and the emotional skill set. At the classroom level, instructors can embed low-stakes, structured participation formats (think–pair–share, rotating spokespersons, anonymous polling) that reduce the perceived cost of speaking (Ratnaningsih, 2025; Noman and Xu, 2023). Explicit norm-setting—articulating that mistakes are expected and valuable—can weaken evaluative threat. Providing process-focused feedback rather than outcome-only praise mitigates perfectionism and fear (Copaja and Vera, 2025; Apriana and Lesi Anggraini, 2025). Peer structures, such as learning communities, mentor systems, or cooperative projects with rotating roles, distribute talk time and cultivate trust, thereby elevating IS and indirectly lowering SA (Sikandar et al., 2023; Dueñas-Casado et al., 2025).

At the individual level, brief cognitive-behavioral micro-interventions can be integrated into curricula: students identify catastrophic thoughts, test them against evidence, and engage in graduated exposure (short statements, then longer answers, then presentations) (Akbari et al., 2025; Wang et al., 2025). Embedding socio-emotional learning (SEL) content—emotion labeling, reappraisal, mindfulness—within first-year seminars or academic skills courses institutionalizes anxiety-coping skills (Guo et al., 2025; Jiang et al., 2025; Becker and Moritz Börnert-Ringleb, 2025).

Institutionally, aligning teaching-development centers and counseling services is crucial. Joint workshops can train faculty to recognize anxiety cues and respond with scaffolding, not sanction (Gough et al., 2025). Reward structures (e.g., teaching awards evaluating inclusivity) incentivize sustained relational practices. Policy-wise, assessment designs should acknowledge multimodal participation (oral, written, digital forums), so that speaking is encouraged but not the sole route to engagement, thereby easing pressure while keeping voice central (Gore et al., 2017).

Finally, technology-mediated classrooms offer both opportunities and risks. Tools like anonymous Q&A platforms can lower entry barriers for anxious students, but overreliance may prevent gradual exposure to public speaking (Ai et al., 2025; Kok et al., 2025). Thus, tech should be used strategically—initially to scaffold participation, and gradually to support students’ transition to more visible forms of contribution (Kumari, 2024), with the understanding that these implications are grounded in correlational findings and should be further tested in intervention studies.

### 5.5 Limitations

Despite the strengths of a multi-indicator latent modeling approach and a multi-site Chinese sample, several limitations constrain the interpretability and generalizability of our findings. First, the cross-sectional design precludes strong causal inference. Although the proposed ordering—from interpersonal satisfaction (IS) to social anxiety (SA) to classroom silence (CS)—is theoretically grounded in social-cognitive logic and supported by prior longitudinal evidence on related constructs, reciprocal and feedback effects remain plausible. Students’ persistent silence could gradually corrode relational networks, thereby lowering subsequent IS and heightening SA; conversely, high IS might accumulate precisely because students were already more willing to speak. Without temporal data, such dynamics cannot be disentangled.

Second, measurement granularity is limited. CS was captured through a mix of frequency indicators and context-specific behaviors, yet silence is a heterogeneous phenomenon that includes reflective, strategic, and avoidance-based forms. Lumped operationalization risks conflating adaptive quiet with anxiety-driven muteness. Similarly, IS was measured via self-reports of satisfaction across a few relational domains; these ratings tell us little about network structure (density, reciprocity) or power asymmetries in classrooms that might independently shape anxiety and voice.

Third, all focal constructs and personality covariates were assessed via self-report. Although statistical checks suggested that common-method bias was not dominant, shared-method variance and social desirability bias remain plausible, particularly for anxiety and silence measures. Self-reported silence may diverge from actual behavioral participation, which limits the precision with which we can draw conclusions about observable classroom behavior. Triangulation with behavioral traces, peer nominations, or instructor ratings would sharpen construct validity. In addition, the personality controls were measured with single items, which provide only coarse indicators of underlying traits and are likely to have lower reliability than multi-item scales.

Fourth, the sampling frame was geographically and institutionally constrained. Although our sampling spanned multiple majors, all participants were drawn from public, teaching-focused universities in a single Chinese province. Class sizes, pedagogical philosophies, and assessment regimes differ across elite research universities, teaching-oriented institutions, and vocational colleges; these macro-contexts likely moderate the micro-level pathways observed here. Finally, some analytic decisions warrant caution. Treating ordinal Likert scales as continuous indicators in SEM is common practice, but nuances may be lost relative to polychoric approaches; likewise, model fit indices—while acceptable—cannot guarantee that alternative theoretically plausible models would not fit equally well. Collectively, these limitations do not negate our conclusions but delineate their boundary conditions and suggest avenues for methodological refinement.

### 5.6 Future research directions

Future work should move beyond static snapshots toward designs that illuminate the temporal, contextual, and mechanistic complexity of the IS–SA–CS nexus. Longitudinal and cross-lagged panel studies can test bidirectionality: Do improvements in interpersonal climates genuinely precede declines in anxiety and silence, or do students who break their silence subsequently strengthen relational bonds that further reduce fear? Intensive methods such as experience-sampling, diary studies, or learning analytics could capture within-person fluctuations in anxiety and voice across class sessions, revealing how momentary relational cues (e.g., a teacher’s supportive comment, a peer’s affirmation) are linked to speaking versus remaining silent. Given that self-reported silence may diverge from observed participation, future studies should incorporate classroom video coding or audio-based participation logs to obtain behavioral indicators alongside self-reports.

Conceptual refinement is equally crucial. Distinguishing functional silence (reflective, strategic, culturally normative) from dysfunctional silence (avoidant, defensive) requires mixed-methods strategies that combine validated scales with qualitative interviews or focus groups, to prevent over-pathologizing quiet behaviors that may serve pedagogical purposes in certain contexts. On the relational side, social network analysis can map structural features of classroom communities—who sits at the periphery, who anchors subgroups—and test whether positional attributes mediate or moderate anxiety’s effect on voice. The affective pathway also invites elaboration: incorporating emotional intelligence, specific emotion-regulation strategies (reappraisal vs. suppression), and physiological indicators could clarify why some low-IS students still speak and why others remain silent despite high IS (Kahraman, 2022; Zhao et al., 2024).

Experimental and quasi-experimental intervention studies are needed to move from association to stronger causal claims. Randomized trials of relational interventions and anxiety-focused modules could identify which lever—or combination of levers—is most strongly associated with reductions in anxiety-driven silence. Multi-level SEM would allow researchers to model classroom or institution-level moderators (norms, assessment culture) on top of individual processes, while cross-cultural comparisons could differentiate mechanisms that are culturally general from those bound to Chinese or broader East Asian contexts. Finally, emerging hybrid and AI-mediated learning environments pose new questions: Will online discussion boards or anonymous polling tools attenuate or exacerbate anxiety-driven silence? Do algorithmic feedback systems change how students read evaluative threat? Experience-sampling and mixed-methods designs will be particularly valuable for addressing these questions in the rapidly evolving ecologies of higher education.

## 6 Conclusion

This study identified a pattern of associations in which higher interpersonal satisfaction is linked to lower social anxiety, which in turn is associated with lower levels of classroom silence among undergraduates in Chinese universities. Using structural equation modeling, we found evidence for a substantial indirect association from interpersonal satisfaction to classroom silence via social anxiety, alongside a smaller direct association, yielding an integrated socio-emotional account of student reticence that centers relational and affective processes. Conceptually, the findings extend social-cognitive accounts by specifying how environmental (relational) conditions are connected to interpersonal (affective) states and, in turn, to communicative behavior, while also qualifying cultural narratives that attribute silence primarily to Confucian norms.

Practically, the results suggest that reducing anxiety-driven silence may require a dual focus on (a) cultivating psychologically safe, relationally supportive classroom climates and (b) embedding light-touch socio-emotional and cognitive coping activities into courses so that students can better manage evaluative fears. Such strategies can be incorporated into ordinary teaching practice without turning every class into a formal intervention.

Methodologically, despite the strengths of a latent-variable approach and multi-indicator measurement, the cross-sectional design precludes strong causal claims and does not allow us to observe potential reciprocal dynamics between relationships, anxiety, and speaking behavior. Future longitudinal, multilevel, and mixed-method research—especially work that distinguishes functional from dysfunctional silence and combines self-report with behavioral indicators—will be needed to test the directionality and contextual boundaries of the IS–SA–CS pathways more rigorously. By mapping a relational–affective route to silence, this study offers a theoretically coherent and practically usable framework for thinking about how to foster classrooms where speaking is experienced as a routine part of collaborative learning rather than a high-stakes test of courage.

## Data Availability

The raw data supporting the conclusions of this article will be made available by the authors, without undue reservation.

## References

[ref1] Ai Z. Yuan D. Dong R. Li Y. Zhou S. (2025). Revealing the impact of teaching methods on anxiety among college students through a bibliometric study. Front. Psychol. 16:1558313. doi: 10.3389/fpsyg.2025.1558313, 40420987 PMC12104304

[ref2] Akbari A. Torabizadeh C. Nick N. Setoodeh G. Ghaemmaghami P. (2025). The effects of training female students in emotion regulation techniques on their social problem-solving skills and social anxiety: a randomized controlled trial. Child Adolesc. Psychiatry Ment. Health 19:3. doi: 10.1186/s13034-025-00860-1, 39844242 PMC11756156

[ref3] Almadina Rakhmaniar (2023). Eksplorasi Pengaruh Kecemasan Sosial Terhadap Gaya Komunikasi Pada Mahasiswa: Pendekatan Grounded Theory. TUTURAN: Jurnal Ilmu Komunikasi, Sosial Dan Humaniora 1, 80–94. doi: 10.47861/tuturan.v1i1.1119

[ref4] Apriana Lesi Anggraini (2025). Fostering the students’ speaking ability through communicative approach of participation point system. Esteem J. English Educ. Study Programme 8, 439–445. doi: 10.31851/esteem.v8i1.17804

[ref5] Archbell K. A. Coplan R. J. (2021). Too anxious to talk: social anxiety, academic communication, and students’ experiences in higher education. J. Emot. Behav. Disord. 30, 273–286. doi: 10.1177/10634266211060079

[ref6] Babanoğlu M. P. (2025). Decoding the relationship between emotional intelligence and foreign language anxiety: a systematic review and meta-analysis. Lang. Teach. Res.:13621688251345726. doi: 10.1177/13621688251345726

[ref7] Bandura A. (1986). Social foundations of thought and action: A social cognitive theory. Englewood Cliffs, NJ, US: Prentice-Hall, Inc.

[ref8] Bauth M. F. Antonio Paulo Angélico Oliveira D. C. R. (2019). Association between social skills, sociodemographic factors and self-statements during public speaking by university students. Temas Psicol. 27, 677–692. doi: 10.9788/TP2019.3-06

[ref9] Becker S. J. Moritz Börnert-Ringleb (2025). Stress and anxiety in schools: a multilevel analysis of individual and class-level effects of achievement and competitiveness. Front. Educ. 9:1519161. doi: 10.3389/feduc.2024.1519161

[ref10] Bentler P. M. Chih-Ping Chou (1987). Practical issues in structural modeling. Sociol. Methods Res. 16, 78–117. doi: 10.1177/0049124187016001004

[ref11] Bi T. Hui Kou Qinhong Xie Jie Dong (2022). Mediating roles of social anxiety and interpersonal distress in the relationship between mobile phone addiction and loneliness. J. Psychol. Afr. 32, 487–493. doi: 10.1080/14330237.2022.2121058

[ref12] Bittel K. M. O’Briant K. Y. Ragaglia R. M. Buseth L. Murtha C. Yu J. . (2023). Associations between social cognitive determinants and movement-related behaviors in studies using ecological momentary assessment methods: systematic review. JMIR Mhealth Uhealth 11:e44104. doi: 10.2196/44104 37027185 PMC10131703

[ref13] Bolsoni-Silva A. T. Loureiro S. R. (2014). The role of social skills in social anxiety of university students. Paidéia (Ribeirão Preto) 24, 223–232. doi: 10.1590/1982-43272458201410

[ref14] Brislin R. W. (1970). Back-translation for cross-cultural research. J. Cross-Cult. Psychol. 1, 185–216. doi: 10.1177/135910457000100301

[ref15] Caballo V. E. Salazar I. C. Irurtia Muñiz M. J. Olivares Olivares P. J. Olivares Rodríguez J. (2014). Relación de Las Habilidades Sociales Con La Ansiedad Social y Los Estilos/Trastornos de La Personalidad. *Behavioral Psychology/Psicología Conductual* 22, 401–422.

[ref16] Chan Simon To Keung Smith G. D. (2024). Strategies for enhancing Chinese students’ engagement in a large class learning environment: an interpretative phenomenological approach. Nurse Educ. Pract. 78:104023. doi: 10.1016/j.nepr.2024.104023 38909458

[ref17] Chen C. Bian F. Zhu Y. (2023). The relationship between social support and academic engagement among university students: the chain mediating effects of life satisfaction and academic motivation. BMC Public Health 23:2368. doi: 10.1186/s12889-023-17301-3, 38031093 PMC10688496

[ref18] Chen J. Ye B. Miao H. (2025). Configuration analysis of negative silence in college classroom based on FSQCA method. Sci. Rep. 15:1. doi: 10.1038/s41598-025-09608-5, 40634503 PMC12241655

[ref19] Choi B. (2025). Exploring the dynamics of social-emotional competencies, fear of failure in learning, and engagement in online learning. Am. J. Distance Educ. 39, 456–472. doi: 10.1080/08923647.2025.2489192

[ref20] Collie R. J. Martin A. J. Papworth B. Ginns P. (2016). Students’ interpersonal relationships, personal best (PB) goals, and academic engagement. Learn. Individ. Differ. 45, 65–76. doi: 10.1016/j.lindif.2015.12.002

[ref21] Copaja C. A. Y. Vera J. R. E. (2025). Neuroeducation strategies that promote participation in the classroom. A belief from the experience of university students in Perú. Edelweiss App. Sci. Technol. 9, 1173–1181. doi: 10.55214/25768484.v9i1.4367

[ref22] De Souza Borba C. De Albuquerque Hayasida N. M. Machado Lopes F. (2020). Ansiedade Social e Habilidades Sociais Em Universitários. Rev. Psicol. Em Pesq. 13, 119–137. doi: 10.34019/1982-1247.2019.V13.27052

[ref23] Demir Ö. Cinar M. Keskin S. (2023). Participation style and social anxiety as predictors of active participation in asynchronous discussion forums and academic achievement. Educ. Inf. Technol. 28, 1–22. doi: 10.1007/s10639-022-11517-3, 36819982 PMC9933033

[ref24] Dos Santos B. R. Sarriera J. C. Bedin L. M. (2019). Subjective well-being, life satisfaction and interpersonal relationships associated to socio-demographic and contextual variables. Appl. Res. Qual. Life 14, 819–835. doi: 10.1007/s11482-018-9611-6

[ref25] Dueñas-Casado C. Falla D. Ortega-Ruiz R. Romera E. M. (2025). Moral disengagement in primary school children involved in cyberbullying, bullying, and cybergossip. Soc. Psychol. Educ. 28:85. doi: 10.1007/s11218-025-10042-8

[ref26] Gore J. Lloyd A. Smith M. Bowe J. Ellis H. Lubans D. (2017). Effects of professional development on the quality of teaching: results from a randomised controlled trial of quality teaching rounds. Teach. Teach. Educ. 68, 99–113. doi: 10.1016/j.tate.2017.08.007

[ref27] Gough L. Miranda R. Hemm M. Norman L. Jara B. (2025). Faculty perceptions of a professional development program for developing CUREs and promoting inclusive and equitable teaching. J. Microbiol. Biol. Educ. 26, e0021524–e0021524. doi: 10.1128/jmbe.00215-24, 39804063 PMC12020812

[ref28] Guo Y. Xu B. Lyu B. Chen C. (2025). How social and emotional skills affect Chinese college student engagement in high-impact educational practices: a moderated mediation model. Stud. High. Educ. 50, 27–46. doi: 10.1080/03075079.2024.2328828

[ref29] Han X. Zhao S.-q. Ge P. Liu Y. Li Q. Y. Wang Y. N. . (2025). Subthreshold anxiety in Chinese college students: prevalence, gender differences, and correlates. BMC Psychol. 13:1. doi: 10.1186/s40359-025-03084-2, 40676708 PMC12273348

[ref30] Hassani S. (2024). Fostering social-emotional competencies to improve social functioning, social inclusion, and school well-being: results of a cluster non-randomized pilot study. Ment. Health Prev. 36:200365. doi: 10.1016/j.mhp.2024.200365

[ref31] Hu J. (2021). Toward the role of EFL/ESL students’ silence as a facilitative element in their success. Front. Psychol. 12:737123. doi: 10.3389/fpsyg.2021.737123, 34489839 PMC8418106

[ref32] Hu L.-t. Bentler P. M. (1999). Cutoff criteria for fit indexes in covariance structure analysis: conventional criteria versus new alternatives. Struct. Equ. Model. 6, 1–55. doi: 10.1080/10705519909540118

[ref33] Hu L. Wang Y. (2023). The predicting role of EFL teachers’ immediacy behaviors in students’ willingness to communicate and academic engagement. BMC Psychology 11:318. doi: 10.1186/s40359-023-01378-x, 37805631 PMC10559511

[ref34] Jaffee S. R. Hanscombe K. B. Haworth C. M. A. Davis O. S. P. Plomin R. (2012). Chaotic homes and children’s disruptive behavior: a longitudinal cross-lagged twin study. Psychol. Sci. 23, 643–650. doi: 10.1177/0956797611431693, 22547656 PMC3494454

[ref35] Jia M. Cheng J. (2024). Effect of teacher social support on students’ emotions and learning engagement: a U.S.-Chinese classroom investigation. Humanit. Soc. Sci. Commun. 11:158. doi: 10.1057/s41599-024-02634-0

[ref36] Jiang X. Zhang A. Zhang Q. (2025). The effects of mindfulness-based intervention on social anxiety, mindfulness, intolerance of uncertainty and emotion dysregulation——a serial multiple mediating structural equation model. Curr. Psychol. 44, 2634–2647. doi: 10.1007/s12144-025-07362-5

[ref37] John O. Naumann L. Soto C. (2008). Paradigm shift to the integrative Big Five trait taxonomy: History, measurement, and conceptual issues. In O. P. John, R. W. Robins, & L. A. Pervin (Eds.), Handbook of personality: Theory and research (3rd ed., pp. 114–158). New York, NY, USA: Guilford Press.

[ref38] Kahraman M. (2022). Investigating the relationship between emotional intelligence and social anxiety levels of university students. Int. J. Psychol. Educ. Studies 9, 1121–1132. doi: 10.52380/ijpes.2022.9.4.688

[ref39] Kang C. Liu J. Chen X. (2025). The impact of interpersonal trust among university students on participation in extracurricular activities: a chain mediation model. BMC Psychol. 13:1. doi: 10.1186/s40359-025-02957-w, 40474261 PMC12139095

[ref40] Kelly S. Foresman G. Winchester D. (2024). Instructor communicative behaviors as cultivators of students’ task value. South Commun. J. 89, 199–211. doi: 10.1080/1041794X.2024.2389814

[ref41] Kok X.-F. K. Pua C. Y. Puah S. Devilly O. Z. Wang P. C. Chua E. C.-P. (2025). The mediating role of student engagement in the relationship between teacher and digital support and learner satisfaction in blended learning environments at higher education. Br. Educ. Res. J. 51, 1313–1341. doi: 10.1002/berj.4123

[ref42] König J. Santagata R. Scheiner T. Adleff A.-K. Yang X. Kaiser G. (2022). Teacher noticing: a systematic literature review of conceptualizations, research designs, and findings on learning to notice. Educ. Res. Rev. 36:100453. doi: 10.1016/j.edurev.2022.100453

[ref43] Krull J. Urton K. Kulawiak P. R. Wilbert J. Hennemann T. (2022). Social-relational classroom climate and its link to primary students’ behavioral problems. Empirische Sonderpadagogik 14, 154–175. doi: 10.25656/01:25769

[ref44] Kumari N. (2024). Student engagement through emotional and social intelligence – an apporach. Int. Res. J. Advanced Eng. Manage. (IRJAEM) 2, 2980–2983. doi: 10.47392/irjaem.2024.0440

[ref45] Langen I. Stamov Roßnagel C. (2023). East is east: Socratic classroom communication is linked to higher stress in students from Confucian heritage cultures. Heliyon 9:e15748. doi: 10.1016/j.heliyon.2023.e15748, 37251875 PMC10209327

[ref46] Larrazabal M. A. Wang Z. Rucker M. Toner E. R. Boukhechba M. Teachman B. . 2025. “Understanding state social anxiety in virtual social interactions using multimodal wearable sensing indicators.” 2025 IEEE international conference on smart computing (SMARTCOMP), 162–169.

[ref47] Lee J. Waldeck D. Holliman A. Banerjee M. Tyndall I. (2022). Feeling socially anxious at university: an interpretative phenomenological analysis. Qual. Rep. 27, 897–919. doi: 10.46743/2160-3715/2022.5270

[ref48] Li Q. Li N. (2023). The effect of social anxiety on prosocial behavior of college students: the mediating role of interpersonal security and the moderating role of basic psychological need satisfaction. Durham, NC, USA: Research Square (a Springer Nature company). doi: 10.21203/rs.3.rs-3171924/v1

[ref49] Li B. Yu J. Sun L. Yang H. (2024). Impact of active learning instruction in blended learning on students’ anxiety levels and performance. Front. Educ. 9:1332778. doi: 10.3389/feduc.2024.1332778

[ref50] Li X. Zhu Y. Shi X. (2024). Interpersonal sensitivity as a mediator linking interpersonal stressors and social anxiety: longitudinal mediation analysis using parallel process latent growth curve modeling. J. Affect. Disord. 351, 172–178. doi: 10.1016/j.jad.2024.01.218, 38296055

[ref51] Liu C. Elhai J. D. Montag C. Yang H. (2024). Social anxiety and attentional Bias to negative emotional information: the relationship and intervention. BMC Psychiatry 24:508. doi: 10.1186/s12888-024-05938-2, 39020338 PMC11256405

[ref52] Liu M. Jackson J. (2008). An exploration of Chinese EFL learners’ unwillingness to communicate and foreign language anxiety. Mod. Lang. J. 92, 71–86. doi: 10.1111/j.1540-4781.2008.00687.x

[ref53] Lu R. Zhou J. Zhao S. Ou W. Ge J. Yang Y. . (2025). The relationship of anxiety, benefit finding and academic engagement among Chinese college students in different stages of COVID-19: a repeated cross-sectional pilot study. Curr. Psychol. 44, 3178–3196. doi: 10.1007/s12144-025-07281-5

[ref54] Ma J. Lin B. (2022). The impact of college students’ social anxiety on interpersonal communication skills: a moderated mediation model. Sci. Soc. Res. 4, 101–108. doi: 10.26689/ssr.v4i6.3993

[ref55] Ma L. Xia J. (2025). Teacher-student relationships as a buffer: mitigating anxiety’s impact on willingness to communicate across genders. Int. J. Appl. Linguist.:ijal.12792. doi: 10.1111/ijal.12792

[ref56] Maher K. King J. (2020). Observing anxiety in the foreign language classroom: student silence and nonverbal cues. J. Psychol. Language Learn. 2, 116–141. doi: 10.52598/jpll/2/1/6

[ref57] Maher K. King J. (2022). ‘The silence kills me.’: ‘Silence’ as a trigger of speaking-related anxiety in the English-medium classroom. Engl. Teach. Learn. 46, 213–234. doi: 10.1007/s42321-022-00119-4

[ref58] Maher K. King J. (2023). Language anxiety and learner silence in the classroom from a cognitive-behavioral perspective. Annu. Rev. Appl. Linguist. 43, 105–111. doi: 10.1017/s0267190523000077

[ref59] Markus H. R. Kitayama S. (1991). Culture and the self: implications for cognition, emotion, and motivation. Psychol. Rev. 98, 224–253. doi: 10.1037/0033-295X.98.2.224

[ref60] McBeath M. Drysdale M. T. B. Bohn N. (2018). Work-integrated learning and the importance of peer support and sense of belonging. Education + Training 60, 39–53. doi: 10.1108/ET-05-2017-0070

[ref61] Mi Y. Wang Z. Peng L. Zhang C. Xu H. (2025). Exploring the impact of interpersonal sensitivity on anxiety symptoms: the mediating role of psychological capital and social support among nursing students. BMC Psychol. 13:290. doi: 10.1186/s40359-025-02621-3, 40121470 PMC11930005

[ref62] Myoung E. Liou P.-Y. (2025). Open classroom climate in international large-scale assessments: individual construct or climate construct? SAGE Open 15:21582440251323560. doi: 10.1177/21582440251323560

[ref63] Noman M. Xu R. (2023). Breaking the silence: exploring the challenges of oral participation faced by Chinese undergraduate students in a Sino-US university in China. Asian Educ. Dev. Stud. 12, 275–286. doi: 10.1108/aeds-04-2023-0036

[ref64] Okazaki S. (1997). Sources of ethnic differences between Asian American and white American college students on measures of depression and social anxiety. J. Abnorm. Psychol. 106, 52–60. doi: 10.1037/0021-843X.106.1.52, 9103717

[ref65] Özdemir O. Seçkin H. (2025). Exploring foreign language anxiety in higher education: multifaceted insights into causes, impacts, and coping strategies. Soc. Sci. Humanities Open 11:101364. doi: 10.1016/j.ssaho.2025.101364

[ref66] Pentón Herrera L. J. (2024). Into the void: re-envisioning silence in foreign language education through a socio-emotional learning lens. Neofilolog 63/2, 293–312. doi: 10.14746/n.2024.63.2.4

[ref67] Phylactou P. Konstantinou N. Ester E. F. (2024). Advancing working memory research through Cortico-cortical transcranial magnetic stimulation. Front. Hum. Neurosci. 18:1504783. doi: 10.3389/fnhum.2024.1504783, 39717149 PMC11663928

[ref68] Ratnaningsih P. W. (2025). Students’ perceptions on small group discussion and writing skill in English class. Esteem J. English Educ. study Programme 8, 446–452. doi: 10.31851/esteem.v8i1.17792

[ref69] Rhemtulla M. Brosseau-Liard P. É. Savalei V. (2012). When can categorical variables be treated as continuous? A comparison of robust continuous and categorical SEM estimation methods under suboptimal conditions. Psychol. Methods 17, 354–373. doi: 10.1037/a0029315, 22799625

[ref70] Scanlon C. L. Del Toro J. Wang M.-T. (2020). Socially anxious science achievers: the roles of peer social support and social engagement in the relation between adolescents’ social anxiety and science achievement. J. Youth Adolesc. 49, 1005–1016. doi: 10.1007/S10964-020-01224-Y, 32206958

[ref71] Sezin R. Sal S. (2025). Evaluation of speaking self-efficacy and social anxiety in secondary school students. Educ. Sci. 50, 129–139. doi: 10.15390/EB.2025.13011

[ref72] Sikandar A. Zamir S. Jabeen S. (2023). Exploring factors underlying intermediate students’ silence in English as second language classroom: a phenomenological case study. Int. J. Soc. Sci. Entrepreneurship 3, 20–44. doi: 10.58661/ijsse.v3i4.210

[ref73] Summerfeldt L. J. Kloosterman P. H. Antony M. M. Parker J. D. A. (2006). Social anxiety, emotional intelligence, and interpersonal adjustment. J. Psychopathol. Behav. Assess. 28, 57–68. doi: 10.1007/S10862-006-4542-1

[ref74] Sun M. Piao M. Jia Z. (2024). The impact of alexithymia, anxiety, social pressure, and academic burnout on depression in Chinese university students: an analysis based on SEM. BMC Psychology 12:757. doi: 10.1186/s40359-024-02262-y, 39695783 PMC11657414

[ref75] Üstündağ A. Haydaroğlu S. Sayan D. Güngör M. (2025). The relationship between social anxiety levels and effective communication skills of adolescents participating in sports. Sci. Rep. 15:15724. doi: 10.1038/s41598-025-00800-1, 40325045 PMC12053575

[ref76] Wang N. Huang S. Cai J. Huang R. Geng H. (2025). How the brain memorizes the world from others’ perspectives: investigating Allocentric encoding of object features during perspective taking. BMC Psychology 13:691. doi: 10.1186/s40359-025-03022-2, 40597457 PMC12219340

[ref77] Wang H. Zhang X. Jin Y. Ding X. (2024). Examining the relationships between cognitive load, anxiety, and story continuation writing performance: a structural equation modeling approach. Humanit. Soc. Sci. Commun. 11:1297. doi: 10.1057/s41599-024-03840-6

[ref78] Xethakis L. J. Rupp M. Plummer B. R. B. (2024). Why students feel anxious in group work: an investigation into students’ perspectives on the sources of their anxious experiences in group work. Front. Educ. 9:1461747. doi: 10.3389/feduc.2024.1461747

[ref79] Xu F. Yang Y. Chen J. Zhu A.-X. (2022). Behind the silence of the professional classroom in universities: formation of cognition-practice separation among university students—a grounded theory study in China. Int. J. Environ. Res. Public Health 19:21. doi: 10.3390/ijerph192114286, 36361167 PMC9658448

[ref80] Xu J. Zhou S. (2024). Unraveling the interplay between social-emotional need satisfaction and self-regulated learning: longitudinal evidence from university students in China’s EMI programs. Lang. Teach. Res.:13621688241304875. doi: 10.1177/13621688241304875

[ref81] Yang Y. Cui Y. Yao S. (2024). Teacher support, grit and L2 willingness to communicate: the mediating effect of foreign language enjoyment. BMC Psychology 12:383. doi: 10.1186/s40359-024-01877-5, 38982544 PMC11232161

[ref82] Yep B. L. W. Tan T. K. Fung F. M. (2023). How partial anonymity may reduce students’ anxiety during remote active learning─a case study using clubhouse. J. Chem. Educ. 100, 459–468. doi: 10.1021/acs.jchemed.2c00051

[ref83] Zhang L. Zhang Y. (2018). Pilot study of the influence of social anxiousness on students’ classroom interactions among Chinese undergraduates. J. Glob. Educ. Res. 2, 60–70. doi: 10.5038/2577-509X.2.2.1046

[ref84] Zhao Y. Sang B. Ding C. (2024). How emotional intelligence influences students’ life satisfaction during university lockdown: the chain mediating effect of interpersonal competence and anxiety. Behav. Sci. 14:1059. doi: 10.3390/bs14111059, 39594360 PMC11591228

[ref85] Zou Yongshan. 2024. “The impact of social anxiety on college students. SHS web of conferences 193: 02010.

